# Sampling genetic diversity in the sympatrically and allopatrically speciating Midas cichlid species complex over a 16 year time series

**DOI:** 10.1186/1471-2148-7-25

**Published:** 2007-02-20

**Authors:** Paul ME Bunje, Marta Barluenga, Axel Meyer

**Affiliations:** 1 Lehrstuhl für Zoologie und Evolutionsbiologie, Department of Biology, University of Konstanz, Universitätsstraße 10, 78457 Konstanz, Germany

## Abstract

**Background:**

Speciation often occurs in complex or uncertain temporal and spatial contexts. Processes such as reinforcement, allopatric divergence, and assortative mating can proceed at different rates and with different strengths as populations diverge. The Central American Midas cichlid fish species complex is an important case study for understanding the processes of speciation. Previous analyses have demonstrated that allopatric processes led to species formation among the lakes of Nicaragua as well as sympatric speciation that is occurring within at least one crater lake. However, since speciation is an ongoing process and sampling genetic diversity of such lineages can be biased by collection scheme or random factors, it is important to evaluate the robustness of conclusions drawn on individual time samples.

**Results:**

In order to assess the validity and reliability of inferences based on different genetic samples, we have analyzed fish from several lakes in Nicaragua sampled at three different times over 16 years. In addition, this time series allows us to analyze the population genetic changes that have occurred between lakes, where allopatric speciation has operated, as well as between different species within lakes, some of which have originated by sympatric speciation. Focusing on commonly used genetic markers, we have analyzed both DNA sequences from the complete mitochondrial control region as well as nuclear DNA variation at ten microsatellite loci from these populations, sampled thrice in a 16 year time period, to develop a robust estimate of the population genetic history of these diversifying lineages.

**Conclusion:**

The conclusions from previous work are well supported by our comprehensive analysis. In particular, we find that the genetic diversity of derived crater lake populations is lower than that of the source population regardless of when and how each population was sampled. Furthermore, changes in various estimates of genetic diversity within lakes are minimal and provide no evidence for drastic changes during the last 20 years, supporting the hypothesis that the processes which have resulted in rapid speciation are primarily historical. In contrast, there is some evidence for ongoing evolution, particularly selection, in all lakes except crater Lake Masaya, perhaps reflecting the persistence of speciational processes. Importantly, we find that the crater Lake Apoyo population, for which strong evidence of sympatric speciation has been demonstrated, has lower genetic diversity than other crater lakes and the strongest evidence for ongoing selection.

## Background

Over the past several years, largely due to the development of sensitive molecular markers, our knowledge of the factors involved in speciation has grown tremendously. Research into the life history, ecological, and genetic characteristics of speciating lineages has enriched the understanding of the underlying processes that generate diversity [1,2]. Nevertheless, the specific factors that cause particular lineages to diverge genetically remain difficult to generalize beyond the systems in which they have been identified. Useful data to assist in generalizing our understanding of the characteristics of speciating lineages would involve a system in which several biological features are held relatively constant yet some, but not all, members of the diversifying species are undergoing divergent processes and speciation. To this end, we have long investigated the genetic characteristics of the sympatrically and allopatrically speciating Midas cichlid species complex to gain insight into the processes governing divergence [3-6]. Each of these previous studies was based on independent collections, spaced over 16 years, of the various lakes in which these fish live. To test whether it is mostly differences in sampling regime that affect inferences of evolutionary processes, or whether ongoing evolutionary changes can be identified in the lakes, we here both re-analyze these various samples and also include new genetic data from all lakes and time periods to make them directly comparable.

**Figure 1:**
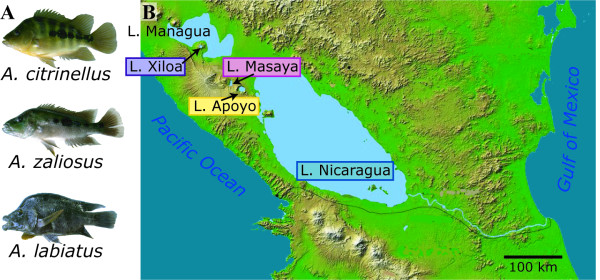
**The Midas cichlid species complex in Nicaragua**. (A) The three described species of the Midas cichlid species complex. *A. citrinellus *is present in all lakes whereas *A. zaliosus*, the arrow cichlid, is the result of sympatric speciation in Lake Apoyo and remains confined to it. *A. labiatus*, the red-devil cichlid is found only in the great lakes and was excluded from this study because there is no evidence that it has participated in the colonization of crater lakes. (B) The four lakes of this study are found in western Nicaragua. Lakes Apoyo, Masaya, and Xiloa are all the result of volcanic processes that have left disconnected craters in which these lakes have formed.

There are currently three described species in the Midas cichlid species complex that have been verified by genetic analysis. These three species, *Amphilophus citrinellus*, *A. labiatus*, and *A. zaliosus*, are distributed in the lakes of western Nicaragua [7,8]. *A. citrinellus *is found in both the great Lakes Managua and Nicaragua as well as several crater lakes in the area (Fig. 1), whereas *A. labiatus *is known only from the two great Lakes and *A. zaliosus *inhabits only one of the small crater lakes, Lake Apoyo [4,9]. Preliminary surveys of several crater lakes have resulted in an increase in the number of putative species [10], though these classifications have not yet been thoroughly verified by more detailed genetic analyses. Whether or not these described species will turn out to be biological species, it is amply clear that the Midas cichlid species complex is highly polymorphic and probably contains several more species [A. Meyer, et al., unpublished data; [11,12]].

Recently, strong evidence for speciational processes in the Midas cichlid species complex has been discovered. Wilson et al. [5] first documented species-level genetic divergence between lakes as well as found some evidence for differentiation between color morphs and trophic morphs within *A. citrinellus*. The evidence for allopatric speciation was supported in the more recent work of Barluenga and Meyer [4], who also found that the black and gold color morphs of both *A. citrinellus *and *A. labiatus *were significantly genetically differentiated in the great Lakes of Managua and Nicaragua.

Most recently, Barluenga et al. [3] have demonstrated the rapid emergence of a new species (*A. zaliosus*) sympatric with its ancestral species within the crater Lake Apoyo. This study used mitochondrial and nuclear genetic data to demonstrate high divergence between *A. citrinellus *and *A. zaliosus *in Lake Apoyo. Furthermore, trophic and morphometric analyses demonstrated that ecological differentiation was significant between these two groups and offered a clear mechanism for adaptively-driven sympatric speciation [6,12]. *A. zaliosus *shows premating behavioral reproductive isolation from *A. citrinellus *[13], a behavior that has apparently evolved during the short history of this lake [ca. 20,000 ybp; [14]]. This may be prove to be one of the primary mechanisms by which not only the diversity of the Midas cichlid complex, but also the spectacular diversity of East African cichlid fishes, has arisen [15-18].

As a part of the ongoing investigation of speciation in Midas cichlids, we have amassed genetic data from samples that were collected over the last 20 years. In addition to the effects that small populations resident in these crater lakes may have on population genetic change through time, the effects of differences in sampling by researchers might have an impact on the estimation of population genetic parameters associated with evolutionary change [19]. Estimates of genetic diversity, and the parameters estimated from these data, can be greatly impacted by factors such as when during the year the samples were collected, where they were collected, and how they were collected, but they may also reflect natural fluctuations in population size and composition, changes in habitat, and changes in selection pressure. The time-series analyzed in the current study means that the effects of the sampling regime on biased samples of diversity are minimized and an unbiased sample of true genetic diversity is more likely attained [20]. Furthermore, we simultaneously analyze changes in population demographics to learn more precisely how these populations may be changing over time. In particular, an Old World cichlid fish, the tilapia (*Oreochromis niloticus*), was introduced into Lake Apoyo in the late 1980's as a foodstuff [21,22]. Any immediate genetic effects that this confamiliar invasive species has had on the native Midas cichlids can therefore be evaluated. Utilizing several samples spread across time allows us to estimate the ongoing effects of both demographic and selective pressures on the genetic diversity of individual populations.

In addition to utilizing previously collected data [3-5], we have increased the size of the data sets for each of the three sample periods in order to make them comparable (Table 1). This includes the collection of six additional microsatellite loci from the 1987 collection, three new microsatellite loci for the 2001 collection, and the sequencing of the second half of the mitochondrial control region sequence for all 1987 samples, and 136 mitochondrial control region sequences from the 2003 collection. In sum, we analyze genetic diversity for 10 microsatellite loci and the full mitochondrial control region for samples from three collections over 16 years using traditional, coalescent-based, and Bayesian population genetic methods. We thereby test the quality of inference from collections at single time points, verify the verity of previous work [3-5], and provide the first view of genetic changes through time in the speciating Midas cichlid species complex.

**Table 1:** Summary of mitochondrial haplotype and microsatellite samples used in this study

	Current Study		Previous Papers		Comments
			
	# mtDNA	# μsats	# mtDNA	# μsats	
			
1987 [5]					
Apoyo	15	24	14	24	one additional individual sequenced
Xiloa	33	51	36	51	three individuals could not be amplified for the entire sequence
Masaya	7	15	6	15	one additional individual sequenced
Nicaragua	20	51	20	51	
*Total*	*75*	*141*	*76*	*141*	
					
2001 [4]					
Apoyo	33	54	33	49	five additional individuals sequenced
Xiloa	18	25	18	25	
Masaya	36	36	36	36	
Nicaragua	58	58	58	58	Only samples comparable to 1987 and 2001 collections were used
*Total*	*145*	*173*	*145*	*168*	
					
2003 [3]					
Apoyo	80	73	80	73	
Xiloa	89	96	89	96	
Masaya	112	118	112	118	
Nicaragua	46	48	46	48	
*Total*	*327*	*335*	*327*	*335*	
					
Total of all samples					
Apoyo	128	151	127	146	
Xiloa	140	172	143	172	
Masaya	155	169	154	169	
Nicaragua	124	157	124	157	
*Total*	*547*	*649*	*548*	*644*	

Shown are the number of individuals sequenced for the mitochondrial control region and the number that were genotyped for 10 microsatellite loci. Differences from previous papers are explained.

## Methods

### Specimen collection

Samples of *A. citrinellus *and *A. zaliosus *were collected from two large ancient lakes and three young crater lakes in Nicaragua in 1987, 2001, and 2003. It has been previously demonstrated that these two species form a monophyletic unit, excluding *A. labiatus *[4]. As *A. labiatus *is only found in the great lakes, we have only analyzed the monophyletic unit represented by the other two species. The freshwater fish fauna of the two large lakes are thought to be approximately 500,000 years old and are believed to have separated from an ancient lake that formed <1 mya [14,23]. The three crater lakes investigated show somewhat different patterns. Crater Lake Xiloa is believed to have at one time been a part of the large ancient lake that also included its neighbor, the great Lake Managua, though the time of its separation is uncertain [24]. The other two crater lakes, Lake Masaya and Lake Apoyo are thought to be much younger, with the age of Lake Apoyo less than 23,000 years [14]. Whole fish or fin clips were preserved in ethanol until subsequent genomic DNA extraction [method described in [25]].

### Haplotype sequencing and microsatellite genotyping

The complete sequence of the mitochondrial control region (826–836 bp) as well as genotypes for ten microsatellite loci were determined for each specimen; Table 1 details the source lakes and collection years. Some of these data were collected and analyzed as a part of previous studies on the pattern of speciation in the Midas cichlid species complex [3-5]. Additional data was generated for this study so that the samples from all three collections could be compared fully. The sequence of the second half of the mitochondrial control region for the 1987 samples was determined following the protocol outlined in Barluenga and Meyer [4]. 75 total individuals were sequenced from 1987 (representing 46 unique haplotypes) [GenBank: EF219198–EF219272]. The 145 sequences from 2001 represent 48 unique haplotypes [GenBank: AY567011–AY567033, AY567036–AY567045, AY567047, AY567049, AY567052, AY567054, AY567057, AY567059–AY567060, AY567063, AY567065, AY567082, AY567091, AY567097, AY567103, AY567109–AY567112, AY567115, AY567119, AY567134, AY567138, AY567146, AY567152, AY567159, AY567175–AY567265]. The 327 sequences from 2003 represent 91 haplotypes [GenBank: DQ229964–DQ230081, GenBank: EF157327–EF157573]. Ten unlinked [3] microsatellite loci were genotyped for these individuals. New data generated for this study include six additional microsatellite loci [Acit6, TmoM7, UNH002, UNH011, UNH012, UNH013; see ref. [3]] for the 1987 samples and three additional loci [UNH011, UNH012, UNH013; see ref. [11]] for the 2001 samples. In sum, a total of 141 individuals were genotyped from 1987, 173 individuals from 2001, and 335 individuals from 2003 (Table 1). Methods for sequencing the mitochondrial DNA (mtDNA) are described in Barluenga and Meyer [4] and methods describing microsatellite genotyping are described in Barluenga and Meyer [4] and Barluenga et al. [3].

### Relationships among populations

Relationships among the populations from different lakes of the Midas cichlid species complex were examined with both the mitochondrial and nuclear datasets. The degree of interpopulational differentiation was measured using pairwise F_ST _estimates of both the mitochondrial haplotypes and microsatellite alleles for each pair of lakes. Significance was tested using 10,000 random permutations of genotypes among populations, implemented in ARLEQUIN v. 2.001 [26], after sequential Bonferroni correction [27].

Discrimination between populations using nuclear loci was also assessed using the Bayesian assignment procedures implemented in the software STRUCTURE v. 2.1 [28]. To identify the likely number of populations within *A. citrinellus*, STRUCTURE was used to assign a probability of assignment of each individual to different genotypic clusters defined by the ten microsatellite loci [29]. We used an admixture model of genetic clustering run for 10^6 ^generations after a burn-in of 10^5 ^generations. We assumed that there were up to seven clusters (*k *= 1 to 7; preliminary analyses with higher values of *k *were highly unlikely) and ran three parallel chains to estimate what number of genetic clusters had the highest probability.

### Differentiation within populations

We measured several population genetic parameters that can help to distinguish between the various forces, including demographic and selective pressures, that might be influencing genetic divergence in this species complex. First, to determine if genetic change was occurring in any of the populations, we estimated deviations from Hardy-Weinberg equilibrium for each of the microsatellite loci in each population. Then, to test for the presence of selective neutrality, several metrics were estimated. Tajima's [30] D, Fu and Li's [31] F* and D*, and Fu's [32] Fs were calculated for haplotype data using DNASP v. 4.10 [33]. These methods take into account the particular apportioning of genetic variation based on a neutral model of evolution. Given similar demographic conditions, when mutations segregate in a biased manner on individual haplotypes within populations, selection can be inferred – potentially as a mechanism resulting in deviations from Hardy-Weinberg equilibrium. We also tested for selective neutrality using the sampling distribution of mtDNA alleles in a population as implemented in the Ewans-Watterson tests of selective neutrality. For this, we used Slatkin's [34,35] exact test of neutrality as implemented in ARLEQUIN. Finally, historic demographic effects such as population size expansion can be modeled using a pairwise mismatch distribution of haplotype sequences [36]. This procedure determines the probability that the observed mismatch distribution comes from a population having undergone recent population growth (i.e. is unimodal) by comparison with a randomized distribution of the observed data using a parametric bootstrap under a model of sudden demographic expansion [36,37]. Because we expect mtDNA mutation to be negligible during our study period, we combined all mtDNA haplotypes for each lake and estimated the mismatch distribution for each in ARLEQUIN.

### Analyses of genetic diversity

For each lake sampled at each time point, standard nucleotide (π) and haplotype (H) diversities [38] were computed for mtDNA haplotypes using ARLEQUIN. These metrics provide an estimate of the mitochondrial genetic diversity present in a population, allowing the observation of changes in genetic diversity through time. Differences in π and H between lakes within years and between years for each lake were tested using one-tailed *t*-tests and significance was assessed following sequential Bonferroni correction [27].

For the microsatellite loci, we calculated average gene diversity (H¯
 MathType@MTEF@5@5@+=feaafiart1ev1aaatCvAUfKttLearuWrP9MDH5MBPbIqV92AaeXatLxBI9gBaebbnrfifHhDYfgasaacH8akY=wiFfYdH8Gipec8Eeeu0xXdbba9frFj0=OqFfea0dXdd9vqai=hGuQ8kuc9pgc9s8qqaq=dirpe0xb9q8qiLsFr0=vr0=vr0dc8meaabaqaciaacaGaaeqabaqabeGadaaakeaadaqdaaqaaiabdIeaibaaaaa@2DD6@) within each population [38]. This metric can be thought of as the expected heterozygosity (*H*_E_) averaged across all ten loci. Whereas we expect these loci to show neutral patterns of evolution, the fact that these loci are unlinked implies that averaging *H*_E _may produce biased estimates of gene diversity associated with unknown gametic phases in individuals [26]. Differences in H¯
 MathType@MTEF@5@5@+=feaafiart1ev1aaatCvAUfKttLearuWrP9MDH5MBPbIqV92AaeXatLxBI9gBaebbnrfifHhDYfgasaacH8akY=wiFfYdH8Gipec8Eeeu0xXdbba9frFj0=OqFfea0dXdd9vqai=hGuQ8kuc9pgc9s8qqaq=dirpe0xb9q8qiLsFr0=vr0=vr0dc8meaabaqaciaacaGaaeqabaqabeGadaaakeaadaqdaaqaaiabdIeaibaaaaa@2DD6@ between lakes within years and between years for each lake were tested using one-tailed *t*-tests and significance was assessed using sequential Bonferroni correction [27]. Finally, allelic richness (N¯A
 MathType@MTEF@5@5@+=feaafiart1ev1aaatCvAUfKttLearuWrP9MDH5MBPbIqV92AaeXatLxBI9gBaebbnrfifHhDYfgasaacH8akY=wiFfYdH8Gipec8Eeeu0xXdbba9frFj0=OqFfea0dXdd9vqai=hGuQ8kuc9pgc9s8qqaq=dirpe0xb9q8qiLsFr0=vr0=vr0dc8meaabaqaciaacaGaaeqabaqabeGadaaakeaadaqdaaqaaiabd6eaobaadaWgaaWcbaGaemyqaeeabeaaaaa@2F19@) was measured for each population at each sampled time point. N¯A
 MathType@MTEF@5@5@+=feaafiart1ev1aaatCvAUfKttLearuWrP9MDH5MBPbIqV92AaeXatLxBI9gBaebbnrfifHhDYfgasaacH8akY=wiFfYdH8Gipec8Eeeu0xXdbba9frFj0=OqFfea0dXdd9vqai=hGuQ8kuc9pgc9s8qqaq=dirpe0xb9q8qiLsFr0=vr0=vr0dc8meaabaqaciaacaGaaeqabaqabeGadaaakeaadaqdaaqaaiabd6eaobaadaWgaaWcbaGaemyqaeeabeaaaaa@2F19@ provides a representative measure of the quantity of genetic diversity present in a population [39]. It is sensitive to demographic changes such that events like a bottleneck are expected to reduce allelic richness across all loci, whereas selection is expected to affect allelic richness at only one or a few loci. Thus, N¯A
 MathType@MTEF@5@5@+=feaafiart1ev1aaatCvAUfKttLearuWrP9MDH5MBPbIqV92AaeXatLxBI9gBaebbnrfifHhDYfgasaacH8akY=wiFfYdH8Gipec8Eeeu0xXdbba9frFj0=OqFfea0dXdd9vqai=hGuQ8kuc9pgc9s8qqaq=dirpe0xb9q8qiLsFr0=vr0=vr0dc8meaabaqaciaacaGaaeqabaqabeGadaaakeaadaqdaaqaaiabd6eaobaadaWgaaWcbaGaemyqaeeabeaaaaa@2F19@ measured through time can effectively identify changes in genetic diversity associated with both extrinsically and intrinsically induced population fluctuations [39]. N¯A
 MathType@MTEF@5@5@+=feaafiart1ev1aaatCvAUfKttLearuWrP9MDH5MBPbIqV92AaeXatLxBI9gBaebbnrfifHhDYfgasaacH8akY=wiFfYdH8Gipec8Eeeu0xXdbba9frFj0=OqFfea0dXdd9vqai=hGuQ8kuc9pgc9s8qqaq=dirpe0xb9q8qiLsFr0=vr0=vr0dc8meaabaqaciaacaGaaeqabaqabeGadaaakeaadaqdaaqaaiabd6eaobaadaWgaaWcbaGaemyqaeeabeaaaaa@2F19@ was measured for each population at each time sample by randomly sampling with replacement 20 alleles for each microsatellite locus and averaging the number of alleles at each locus. This bootstrap procedure was run for 10,000 iterations using POPTOOLS [40] and retained the central 95% of samples as confidence intervals.

## Results

### Interlacustrine differentiation

Significant divergence between lakes was found with both mitochondrial and nuclear markers as measured by F_ST _(Table 2). Of the significant F_ST _values, Lake Apoyo has by far the largest variance partitioning between populations. These high F_ST _values are consistent regardless of the time period from which the samples are taken. Taking all time samples together, Lake Apoyo F_ST_s range from 0.13 to 0.23 for microsatellites and from 0.29 to 0.47 for mtDNA. The lowest F_ST _values are generally between Lake Masaya and Lake Nicaragua, while Lake Xiloa also shows lower levels of divergence from Lake Masaya and Lake Nicaragua. Generally, F_ST _values for all lakes indicate moderate to high levels of interpopulational structuring.

**Table 2:** Pairwise F_ST _values

	Apoyo	Xiloa	Masaya	Nicaragua
	
1987				
Apoyo		0.428**	0.383**	0.256**
Xiloa	0.261**		0.029	0.212**
Masaya	0.182**	0.049**		0.141*
Nicaragua	0.166**	0.040**	0.050**	
				
2001				
Apoyo		0.474**	0.457**	0.298**
Xiloa	0.217**		0.148*	0.036*
Masaya	0.212**	0.066**		0.065**
Nicaragua	0.143**	0.047**	0.058**	
				
2003				
Apoyo		0.499**	0.478**	0.327**
Xiloa	0.228**		0.094**	0.090**
Masaya	0.179**	0.084**		0.092*
Nicaragua	0.146**	0.066**	0.047**	
				
All samples				
Apoyo		0.466**	0.464**	0.290**
Xiloa	0.226**		0.088**	0.060**
Masaya	0.164**	0.062**		0.083**
Nicaragua	0.133**	0.062**	0.023**	

F_ST _values compare differentiation between lakes in each of the time samples and with all samples pooled together. Microsatellite F_ST_s are below the diagonal, mitochondrial DNA Φ_ST_s are above the diagonal. * p < 0.05, after sequential Bonferroni correction; ** p < 0.001, after sequential Bonferroni correction.

**Figure 2:**
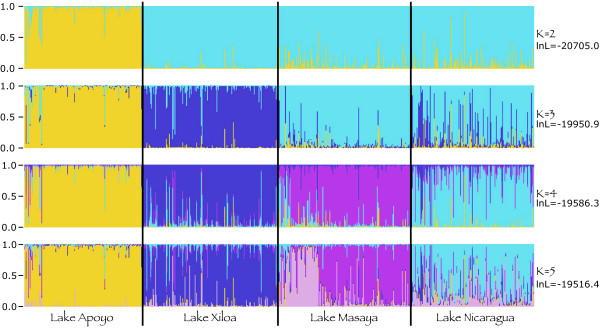
**Results of the analysis for genetic clustering using STRUCTURE**. The lakes from which each individual was collected are indicated below the graph. Colors correspond to the different genetic clusters estimated by the analysis (colors are the same as in Fig. 1), each individual has a probability of being assigned to a given cluster that is proportional to the height of that colored bar, the more uniform the color of the bar, the more probable it is that that individual is entirely composed of genetic material composed of the given structure. The log likelihood of each assumed number of populations (*k *= 2 to 5) is shown.

The model-based clustering method implemented in STRUCTURE found that the most probable number of clusters was *k *= 5 (LnP(D) = -19516.4). This was the case for each of the three independent chains. Figure 2 describes the relationship between these genetic clusters and the lakes from which they were collected. These genetic clusters conform well to expectations based on individuated lakes with limited or no gene flow between them. Additionally, if *k *is allowed to vary, it is apparent that fish from Lake Apoyo form the most distinct genetic cluster, separate from fish in the other lakes at *k *= 2. Following this, at *k *= 3 the fish from Lake Xiloa are found to be distinct, whereas fish from Lake Masaya tend to group strongly with those from Lake Nicaragua. At values of *k *higher than 4, Lakes Nicaragua and Masaya are found to have potentially heterogenous genetic characteristics.

### Intralacustrine differentiation

Measures of deviation from neutrality for mtDNA samples from each lake are described in Table 3. Fu and Li's D* and F* tended to give analogous results, finding Nicaragua to have significantly negative values in all sample years and Lake Apoyo to have significantly negative values in 2001 and 2003. Significance can be strongly affected by sample size (see Table 1), implying that the negative values for D* and F* in 1987 Lake Apoyo samples may not have enough power to provide statistical confidence. This is likely also true for Lake Xiloa samples from 2001, when values of D*, F*, and Fs are all much higher than in the other two collection years. Notably, Lake Masaya has the highest (closest to zero) values of D*, F*, Fs, and D when time points are combined. Lake Xiloa has values that vary through time, but considered together are significantly negative. Fu's Fs statistic is not significant for any sample. However, the highly negative values of some of Fs statistics for all lakes except Masaya indicate at least some deviation from neutrality for these lakes. This result is also found by Tajima's D, where only Lake Masaya has no significantly negative values and has the highest D value in all time periods. Finally, the Ewens-Watterson test of selective neutrality also highlights the deviation present in Lake Apoyo and Lake Nicaragua, with no evidence for non-neutrality in Lake Masaya.

**Table 3:** Tests of selective neutrality in mtDNA sequences

	Fu and Li [31]		Fu [32]	Tajima [30]	Ewens-Watterson [34]	
				
	D*	F*	Fs	D	Observed F	Expected F
				
1987						
Apoyo	-1.627	-1.804	-6.636	-1.422	0.102	0.099
Xiloa	-2.091	-2.399	-12.321	-1.928*	0.118	0.094
Masaya	-0.585	-0.674	-1.447	-0.734	0.184	0.184
Nicaragua	-2.599*	-2.620*	-5.509	-1.423	0.075	0.075
						
2001						
Apoyo	-2.813*	-2.901*	-2.752	-1.733	0.513*	0.293
Xiloa	-0.359	-0.715	-0.491	-1.315	0.549	0.432
Masaya	-0.807	-1.067	-0.122	-1.111	0.235	0.258
Nicaragua	-3.225*	-3.395*	-29.937	-2.199*	0.114**	0.045
						
2003						
Apoyo	-2.560*	-2.847*	-11.478	-2.090*	0.225*	0.108
Xiloa	-1.439	-1.874	-20.654	-1.797*	0.202**	0.080
Masaya	-1.627	-1.951	-5.065	-1.635	0.201	0.170
Nicaragua	-2.978	-3.205	-24.875	-2.177*	0.053*	0.042
						
All years						
Apoyo	-2.398*	-2.767*	-23.969	-2.169*	0.252**	0.078
Xiloa	-3.081*	-3.245*	-43.911	-2.142*	0.142**	0.053
Masaya	-0.835	-1.295	-6.343	-1.452	0.168	0.153
Nicaragua	-3.669*	-3.688*	-88.569	-2.279*	0.059**	0.020

* p < 0.05; ** p < 0.001

Evidence for ongoing evolutionary change in Midas cichlid populations can be found in the deviation from Hardy-Weinberg equilibrium that is present in all lakes. Table 4 describes the observed and expected heterozygosity for each microsatellite in each lake at each time point and for all samples combined. Lake Apoyo has the most loci which show evidence of deviation from Hardy-Weinberg expectations, with multiple loci displaying heterozygote deficiency in 1987, 2003, and the pooled samples. When all samples are pooled, all lakes have several loci that are deficient in heterozygotes, though Lakes Apoyo and Nicaragua have six and five, respectively compared to the three loci that are heterozygote deficient in Lakes Xiloa and Masaya. All lakes show heterozygote deficiency in the 1987 sample while Lake Masaya and Lake Nicaragua show no deviation from expectations in either 2001 or 2003. Notably, the 2001 collection displays no evidence of deviations from Hardy-Weinberg expectations in any lake. These results point to ongoing evolutionary change in the Lake Apoyo population and, to a lesser degree, the Lakes Xiloa and Nicaragua populations (particularly when all samples are pooled), while Lake Masaya does not present strong evidence of deviation from Hardy-Weinberg expectations.

**Table 4:** Deviations from Hardy-Weinberg equilibrium

	Apoyo		Xiloa		Masaya		Nicaragua	
				
Locus	H_O_	H_E_	H_O_	H_E_	H_O_	H_E_	H_O_	H_E_
				
1987								
1	N/A		0.061**	0.210	0.000*	0.587	0.250**	0.491
2	0.458**	0.743	0.551**	0.852	0.714	0.934	0.681**	0.935
3	0.667*	0.914	0.680**	0.879	0.462*	0.905	0.717**	0.908
4	0.292**	0.722	0.560	0.564	0.867	0.752	0.714**	0.906
5	0.067	0.067	0.044	0.066	N/A		0.105	0.126
6	0.583*	0.804	0.778	0.909	1.000	0.867	1.000	0.909
7	0.600	0.683	0.786†	0.778	1.000	0.858	0.737	0.708
8	0.611	0.681	0.412	0.576	0.417	0.598	0.586	0.675
9	0.778	0.656	0.550*	0.881	0.667	0.819	0.750*	0.926
10	0.667	0.721	0.789	0.879	1.000	0.905	0.818	0.945
								
2001								
1	0.037	0.091	0.400	0.404	0.639	0.648	0.690	0.776
2	0.759	0.831	0.720	0.869	0.944	0.896	0.914	0.916
3	0.870	0.899	0.880	0.887	0.861	0.850	0.914	0.938
4	0.538	0.639	0.600	0.583	0.611	0.621	0.839	0.894
5	N/A		N/A		N/A		0.052	0.068
6	0.638	0.746	0.840	0.901	0.771	0.794	0.909	0.906
7	0.404	0.421	0.760	0.750	0.771	0.666	0.655	0.675
8	0.583	0.628	0.200	0.416	0.800	0.673	0.619	0.657
9	0.480	0.454	1.000	0.786	0.588	0.765	0.741	0.880
10	0.627	0.621	0.667	0.894	0.842	0.844	0.909	0.949
								
2003								
1	0.072	0.085	0.260	0.295	0.559	0.593	0.667	0.705
2	0.639	0.712	0.792	0.868	0.864	0.879	0.938	0.915
3	0.845	0.900	0.763*	0.897	0.831	0.883	0.979	0.924
4	0.507*	0.708	0.538*	0.629	0.720	0.793	0.771	0.894
5	N/A		N/A		N/A		0.149	0.159
6	0.686	0.772	0.822	0.853	0.814	0.838	0.773	0.917
7	0.409**	0.652	0.438*	0.599	0.607	0.609	0.488	0.613
8	0.486	0.563	0.568	0.518	0.581	0.663	0.787	0.677
9	0.479	0.565	0.646	0.758	0.788	0.790	0.851	0.895
10	0.603**	0.712	0.844*	0.882	0.754	0.849	0.917	0.945
								
All Pooled								
1	0.049*	0.069	0.224	0.283	0.536	0.598	0.545**	0.704
2	0.653*	0.804	0.712**	0.878	0.869**	0.904	0.850*	0.923
3	0.826	0.911	0.756	0.895	0.808	0.888	0.875	0.930
4	0.483**	0.755	0.554**	0.602	0.710	0.766	0.778**	0.901
5	0.007	0.007	0.012	0.018	N/A		0.098	0.100
6	0.659**	0.879	0.823**	0.904	0.810**	0.881	0.857**	0.935
7	0.429*	0.597	0.539	0.660	0.663	0.640	0.608	0.648
8	0.532	0.594	0.516	0.513	0.597	0.666	0.678	0.669
9	0.518	0.538	0.642	0.781	0.755*	0.787	0.792	0.905
10	0.620**	0.685	0.826	0.882	0.782	0.880	0.886*	0.946

Shown are the observed and expected heterozygosity values under Hardy-Weinberg equilibrium for each microsatellite locus in each lake at each time period. H_O _< H_E_: *p < 0.05, **p < 0.001; after sequential Bonferroni correction. H_O _> H_E_: †p < 0.05, ††p < 0.001; after sequential Bonferroni correction.

**Figure 3:**
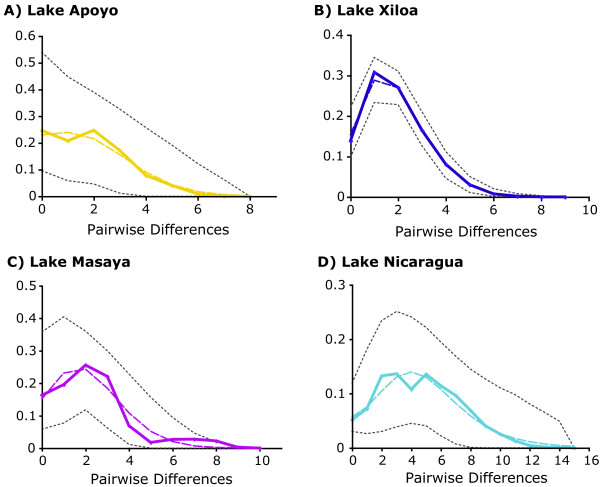
**Results of the pairwise mismatch distribution analyses**. Results are shown using all mtDNA haplotypes from each lake in pooled samples. Observed pairwise differences are shown by the solid line, the distribution under a model of sudden population expansion is shown as a dashed line, and the 95% confidence intervals are shown as gray dotted lines. (A) Lake Apoyo is primarily unimodal, with a small peak at 0 pairwise differences and cannot be distinguished from the distribution under a sudden expansion model. (B) Lake Xiloa is distinctly unimodal and cannot be distinguished from the distribution under a model of sudden population expansion. (C) Lake Masaya is apparently unimodal, though the peak is skewed to a higher pairwise difference than expected under model of sudden population expansion, from which it cannot be distinguished. (D) Lake Nicaragua is multimodal, though it cannot be distinguished from the distribution under a model of sudden population expansion.

Evidence of demographic changes can be deduced from the results of the pairwise mismatch distribution analyses of mtDNA haplotypes (Figure 3). None of the four lakes could be statistically distinguished from the distribution expected under a model of sudden population expansion, indicating that we cannot reject the hypothesis that these populations all result from such an expansion [36,37]. The three crater lakes are all distinctly unimodal, while the large Lake Nicaragua has a pronounced bimodal mismatch distribution. This pattern is consistent with small founding populations in the crater lakes from which the populations have since expanded. Such unimodality is also indicative of close genetic relationships among the members within each crater lake population.

### Genetic diversity

Mitochondrial genetic diversity was measured as the haplotype diversity (H) and nucleotide diversity (π) of Nei [38] for each lake at each collection period as well as being pooled over the period of 16 years (Table 5). Changes in genetic diversity over time were characterized by one-tailed *t*-tests of these values for each lake, significance was assessed using sequential Bonferroni correction [27]. Also, the significance of different levels of mtDNA genetic diversity between lakes was tested for each time frame using the same method (Table 6). Within lakes, the only lake that had significantly different diversity for both H and π at all time comparisons was Lake Apoyo. Lake Nicaragua was the most significantly different lake in interlacustrine comparisons; only H compared to lakes Apoyo and Masaya in 1987 were not significantly different (p < 0.05). Additionally, average genetic diversity (H¯
 MathType@MTEF@5@5@+=feaafiart1ev1aaatCvAUfKttLearuWrP9MDH5MBPbIqV92AaeXatLxBI9gBaebbnrfifHhDYfgasaacH8akY=wiFfYdH8Gipec8Eeeu0xXdbba9frFj0=OqFfea0dXdd9vqai=hGuQ8kuc9pgc9s8qqaq=dirpe0xb9q8qiLsFr0=vr0=vr0dc8meaabaqaciaacaGaaeqabaqabeGadaaakeaadaqdaaqaaiabdIeaibaaaaa@2DD6@) of microsatellite loci was measured for each lake in each time period (Table 5). This result indicates that values of H¯
 MathType@MTEF@5@5@+=feaafiart1ev1aaatCvAUfKttLearuWrP9MDH5MBPbIqV92AaeXatLxBI9gBaebbnrfifHhDYfgasaacH8akY=wiFfYdH8Gipec8Eeeu0xXdbba9frFj0=OqFfea0dXdd9vqai=hGuQ8kuc9pgc9s8qqaq=dirpe0xb9q8qiLsFr0=vr0=vr0dc8meaabaqaciaacaGaaeqabaqabeGadaaakeaadaqdaaqaaiabdIeaibaaaaa@2DD6@ are higher in 2003 than in 1987 for all lakes. As 1987 had the smallest sample size (Table 1), this result could indicate a bias in estimating H¯
 MathType@MTEF@5@5@+=feaafiart1ev1aaatCvAUfKttLearuWrP9MDH5MBPbIqV92AaeXatLxBI9gBaebbnrfifHhDYfgasaacH8akY=wiFfYdH8Gipec8Eeeu0xXdbba9frFj0=OqFfea0dXdd9vqai=hGuQ8kuc9pgc9s8qqaq=dirpe0xb9q8qiLsFr0=vr0=vr0dc8meaabaqaciaacaGaaeqabaqabeGadaaakeaadaqdaaqaaiabdIeaibaaaaa@2DD6@ from small samples since the magnitude of allele frequencies differences depends, in part, on sample size [41].

**Table 5:** Measures of genetic diversity through time in each of the four lakes

	H	π	H¯ MathType@MTEF@5@5@+=feaafiart1ev1aaatCvAUfKttLearuWrP9MDH5MBPbIqV92AaeXatLxBI9gBaebbnrfifHhDYfgasaacH8akY=wiFfYdH8Gipec8Eeeu0xXdbba9frFj0=OqFfea0dXdd9vqai=hGuQ8kuc9pgc9s8qqaq=dirpe0xb9q8qiLsFr0=vr0=vr0dc8meaabaqaciaacaGaaeqabaqabeGadaaakeaadaqdaaqaaiabdIeaibaaaaa@2DD6@
			
1987			
Apoyo	0.962 ± 0.040	0.004 ± 0.002	0.390 ± 0.221
Xiloa	0.909 ± 0.037	0.003 ± 0.002	0.309 ± 0.182
Masaya	0.952 ± 0.096	0.003 ± 0.002	0.412 ± 0.235
Nicaragua	0.974 ± 0.025	0.006 ± 0.004	0.460 ± 0.255
			
2001			
Apoyo	0.502 ± 0.099	0.001 ± 0.001	0.453 ± 0.249
Xiloa	0.477 ± 0.134	0.002 ± 0.001	0.489 ± 0.276
Masaya	0.787 ± 0.042	0.004 ± 0.002	0.498 ± 0.271
Nicaragua	0.843 ± 0.044	0.005 ± 0.003	0.580 ± 0.309
			
2003			
Apoyo	0.839 ± 0.041	0.002 ± 0.002	0.557 ± 0.298
Xiloa	0.840 ± 0.040	0.002 ± 0.001	0.619 ± 0.328
Masaya	0.850 ± 0.038	0.003 ± 0.002	0.678 ± 0.356
Nicaragua	0.975 ± 0.014	0.005 ± 0.003	0.742 ± 0.388
			
All Pooled			
Apoyo	0.771 ± 0.040	0.002 ± 0.001	0.510 ± 0.274
Xiloa	0.870 ± 0.022	0.002 ± 0.001	0.433 ± 0.238
Masaya	0.863 ± 0.021	0.003 ± 0.002	0.585 ± 0.310
Nicaragua	0.926 ± 0.019	0.005 ± 0.003	0.554 ± 0.296

Measures for mtDNA are haplotype diversity (H) and nucleotide diversity (π; Nei, 1987). Also, the average gene diversity (H¯
 MathType@MTEF@5@5@+=feaafiart1ev1aaatCvAUfKttLearuWrP9MDH5MBPbIqV92AaeXatLxBI9gBaebbnrfifHhDYfgasaacH8akY=wiFfYdH8Gipec8Eeeu0xXdbba9frFj0=OqFfea0dXdd9vqai=hGuQ8kuc9pgc9s8qqaq=dirpe0xb9q8qiLsFr0=vr0=vr0dc8meaabaqaciaacaGaaeqabaqabeGadaaakeaadaqdaaqaaiabdIeaibaaaaa@2DD6@) as estimated from microsatellite allelic diversity is given. All values are ± standard deviations. Significance of differences within lakes through time and between lakes in each time period are shown in Table 6 for mtDNA. For microsatellites, the only significant difference for H¯
 MathType@MTEF@5@5@+=feaafiart1ev1aaatCvAUfKttLearuWrP9MDH5MBPbIqV92AaeXatLxBI9gBaebbnrfifHhDYfgasaacH8akY=wiFfYdH8Gipec8Eeeu0xXdbba9frFj0=OqFfea0dXdd9vqai=hGuQ8kuc9pgc9s8qqaq=dirpe0xb9q8qiLsFr0=vr0=vr0dc8meaabaqaciaacaGaaeqabaqabeGadaaakeaadaqdaaqaaiabdIeaibaaaaa@2DD6@ (p < 0.05) within time-samples was between lakes Xiloa and Nicaragua in 1987. H¯
 MathType@MTEF@5@5@+=feaafiart1ev1aaatCvAUfKttLearuWrP9MDH5MBPbIqV92AaeXatLxBI9gBaebbnrfifHhDYfgasaacH8akY=wiFfYdH8Gipec8Eeeu0xXdbba9frFj0=OqFfea0dXdd9vqai=hGuQ8kuc9pgc9s8qqaq=dirpe0xb9q8qiLsFr0=vr0=vr0dc8meaabaqaciaacaGaaeqabaqabeGadaaakeaadaqdaaqaaiabdIeaibaaaaa@2DD6@ was only significantly greater (p < 0.05) in Lake Apoyo when comparing 1987 and 2003, in Lake Xiloa between 1987 and 2003, in Lake Masaya between 1987 and 2003 and 2001 and 2003, and in Lake Nicaragua between 1987 and 2003 and 2001 and 2003.

**Table 6:** Table of significant comparisons in genetic diversity

		Apoyo			Xiloa			Masaya			Nicaragua		
					
		1987	2001	2003	1987	2001	2003	1987	2001	2003	1987	2001	2003
					
Apoyo	1987		††	†	-			-			†		
	2001	**		††		-			††			††	
	2003	**	**				-			-			††
Xiloa	1987	**				-	-	-			††		
	2001		-		**		-		††			††	
	2003			**	**	**				†			††
Masaya	1987	-			-				-	-	†		
	2001		**			**		**		†		†	
	2003			**			-	**	*				††
Nicaragua	1987	-			**			-				-	-
	2001		**			**			**		**		-
	2003			**			**			**	-	**	

Significant differences between time points within lakes and between lakes within time points for haplotype diversity (H; below the diagonal) and nucleotide diversity (π; above the diagonal), see Table 5 for values. Shown are those comparisons that are significant according to a pairwise one-tailed *t*-test, corrected for multiple comparisons by the sequential Bonferroni method. H: *p < 0.05, **p < 0.001; π: †p < 0.05, ††p < 0.001.

**Figure 4:**
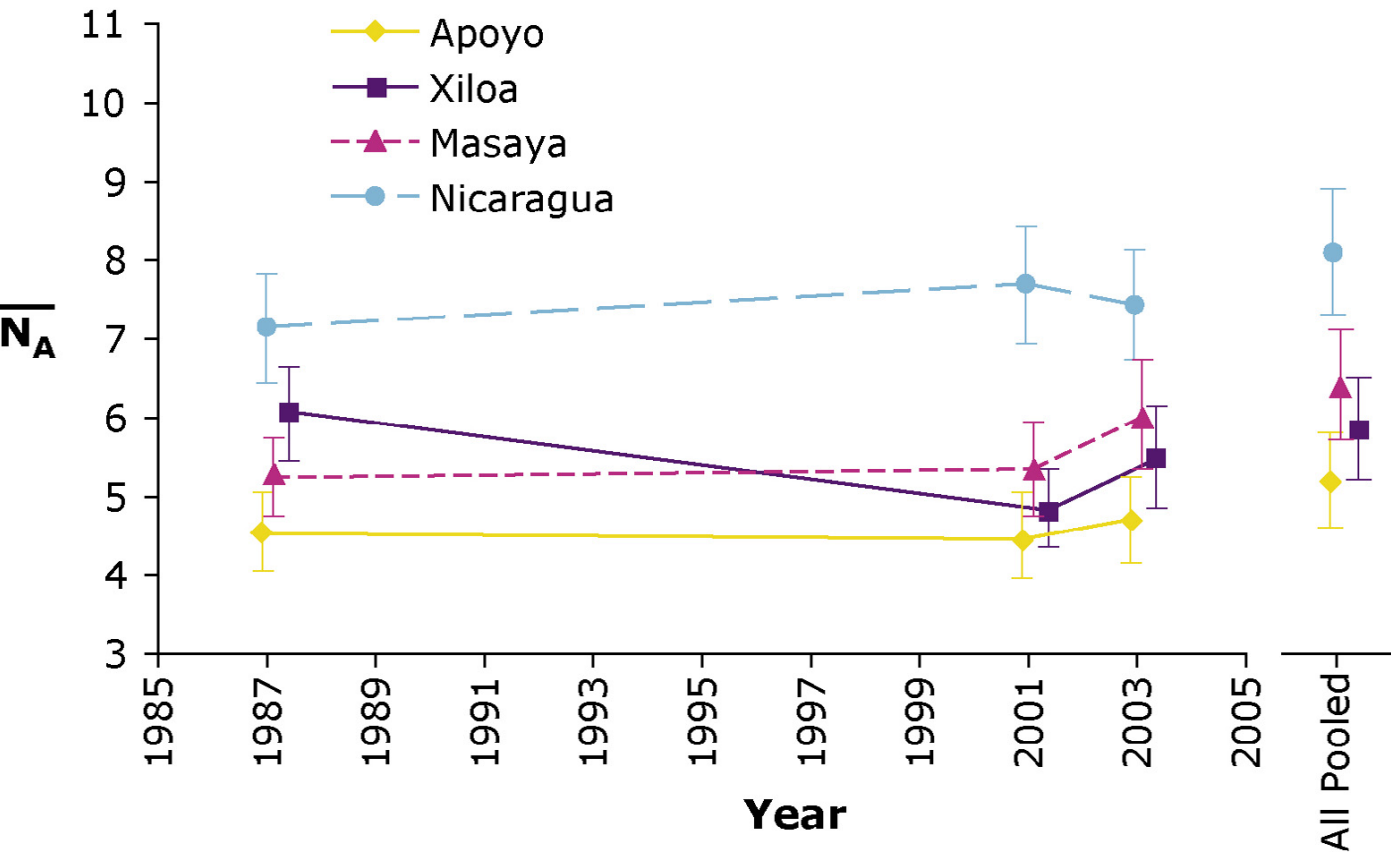
**Average microsatellite allelic richness through time**. Error bars are 95% confidence intervals from the 10,000 bootstrap replicate samples. Also shown are the average allelic diversities for each lake when all genotypes, regardless of sampling year, are pooled. Lake Nicaragua has significantly (p < 0.05) more allelic richness than any of the crater lakes. Only Lake Xiloa has a significant change in allelic richness through time, wherein the 2001 sample is less than 1987.

Finally, to estimate the total amount of nuclear genetic diversity present in each lake, and to determine if this has changed since 1987, we estimated the average allelic richness (N¯A
 MathType@MTEF@5@5@+=feaafiart1ev1aaatCvAUfKttLearuWrP9MDH5MBPbIqV92AaeXatLxBI9gBaebbnrfifHhDYfgasaacH8akY=wiFfYdH8Gipec8Eeeu0xXdbba9frFj0=OqFfea0dXdd9vqai=hGuQ8kuc9pgc9s8qqaq=dirpe0xb9q8qiLsFr0=vr0=vr0dc8meaabaqaciaacaGaaeqabaqabeGadaaakeaadaqdaaqaaiabd6eaobaadaWgaaWcbaGaemyqaeeabeaaaaa@2F19@) for each lake in each time sample. The source population for the crater lake populations, namely Lake Nicaragua, possesses significantly higher allelic richness than any of the crater lakes as evidenced by non-overlapping 95% confidence intervals (Figure 4). Notably, the crater lakes all possess similar levels of genetic diversity, perhaps due to the similar ages of the lakes. However, Lake Apoyo has significantly lower allelic richness than the other lakes according to one-tailed *t*-tests, perhaps reflecting an especially small founder population. Only Lake Xiloa changes significantly through time, with a higher value of N¯A
 MathType@MTEF@5@5@+=feaafiart1ev1aaatCvAUfKttLearuWrP9MDH5MBPbIqV92AaeXatLxBI9gBaebbnrfifHhDYfgasaacH8akY=wiFfYdH8Gipec8Eeeu0xXdbba9frFj0=OqFfea0dXdd9vqai=hGuQ8kuc9pgc9s8qqaq=dirpe0xb9q8qiLsFr0=vr0=vr0dc8meaabaqaciaacaGaaeqabaqabeGadaaakeaadaqdaaqaaiabd6eaobaadaWgaaWcbaGaemyqaeeabeaaaaa@2F19@ in the 1987 sample than in 2001.

## Discussion and conclusion

Despite some variation in estimated parameters of genetic diversity, there is no systematic evidence for population genetic change over the 16 year study period in any of the four lakes sampled. This is significant as it implies that even though the fish were collected by different individuals (though AM participated in all collection trips) and with slightly different localities and methods [see [3-5]], the similar results obtained in all three previous studies are indeed the result of underlying biological processes. Furthermore, this stability over the course of almost 20 generations indicates that any evolutionary forces at work are persistent and apparently not subject to wide variation from one generation to the next, at least not in the standard markers used here. We did not detect any trends in evolutionary pressures through tests of genetic homogeneity, deviations from Hardy-Weinberg equilibrium, or tests of selective neutrality. As a result, our results substantiate the strong evidence for both allopatric and sympatric speciation in the Midas cichlid species complex that has been obtained from different analyses of these independent time samples.

Testing population genetic hypotheses using multiple sampling periods should increase statistical power through multiple independent tests, as well as allow the testing of the hypothesis that the samples being measured are representative of ongoing evolutionary processes [42,43]. Because we find no systematic change in population genetic parameters in these lakes, these results also support the use of single sample collections in inferring evolutionary process. As most population genetic studies do not or cannot sample over long periods of time, it is important to know that in instances when different sampling regimes are used at different times in an evolving group, the evolutionary inferences made based on single collections are likely to be valid.

It should be noted that despite significant differences in haplotype diversity and nucleotide diversity discovered in Lake Apoyo in the three time samples, no trends were apparent that indicate the introduction of tilapia has had an impact on genetic diversity. Allelic richness also stayed consistent for more than 10 years since the introduction of tilapia into this small crater lake. This may be indicative of low or no competition between species [i.e. the occupation of different niche space; [21,22]] or that the introduction of tilapia does not exert measurable selective pressure on the native Lake Apoyo cichlids. This is perhaps unsurprising as observational evidence suggests that the tilapia is not increasing in abundance since its introduction and may be decreasing in abundance [21,22].

Evidence for ongoing evolutionary change within lakes is indicated by the values of Fu and Li's [31] D* and F*, Tajima's [30] D, Fu's [32] Fs, and the Ewens-Watterson test of selective neutrality [34,35]. Whereas the trends identified by D*, F*, D, and Fs may result from either positive selection or population expansion [30,32], deviations detected by the Ewens-Watterson test in both Lake Apoyo and Lake Nicaragua point to selection as a relevant evolutionary force in these systems [35]. In contrast, there is no evidence from these statistics for selection in Lake Masaya (Table 3), where there is no evidence for speciation. This feature is indicative of the strong role selection plays in driving divergence, as other metrics of genetic diversity (Table 5, Fig. 4) indicate that Lake Masaya is similar to crater lakes Xiloa and Apoyo. Furthermore, the mismatch distributions for all lakes indicate that, as expected for the crater lakes in particular, population expansion is a defining feature of the evolutionary change occurring in these localities [37]. The demographic imbalances created by small founding populations in the crater lakes may also account for the deviations from Hardy-Weinberg equilibrium that have been detected. As a result, it is likely that the stochastic sub-sampling of genetic variation associated with founding populations and the persistent small population sizes associated with the crater lake habitats both play a large roll in driving evolutionary change when selection is present. This insight is fully in line with expectations predicting that founder effects and genetic drift associated with small population size can lead to the large phenotypic changes and genetic divergence associated with speciation [44,45].

Lake Apoyo shows the strongest trends associated with both selection and low genetic diversity. It is perhaps not unexpected, then, that the endemic cichlids of this lake also present one of the strongest cases for sympatric speciation yet detected in animals [3,18,46]. Recently, a number of other forms of Midas cichlid from Lake Xiloa were described as distinct species [10,11]. Though this has not been verified through detailed genetic analyses, the similar low values of genetic variation (e.g. Fig. 4), evidence for selection (Table 3), and the mitochondrial genetic homogeneity of the Lake Xiloa flock (Fig. 3), might suggest that if these described species are indeed independent evolutionary lineages, sympatric speciation mechanisms similar to those found in Lake Apoyo may be at work.

Wilson et al. [5] speculated that diversifying forces operate more strongly early in the history of a colonizing population. The combined effects of open niche space, small population size, biased genetic sampling, and genetic drift are expected to decrease as time passes, thus decreasing the rate at which evolutionary change occurs [42]. The results for Lake Xiloa and, especially, Lake Apoyo support such a model. As noted above, Lake Apoyo is the youngest of these crater lakes at <23,000 years old [14]. Lake Masaya is probably not much older [4]. So the sympatric speciation that has produced *A. zaliosus *from a founding population of *A. citrinellus *from Lake Nicaragua may be the clear result of strong diversifying pressures including selection and drift, during the short existence of the population [3]. Though the Midas cichlid fauna of Lake Xiloa is probably much older as suggested by the mismatch analysis (Fig. 3), it is also possible that the several described species in this lake have arisen as a result of its unique founding characteristics. These multiple species may be the result of diversification that began shortly after colonization and has simply had more time to resolve into independent lineages. Alternatively, unique abiotic factors such as increased volcanism in Lake Masaya or Lake Xiloa [14,24], may cause periodic bottleneck events or create unique ecological characteristics that affect the tempo and mode of speciation. Additionally, Lake Xiloa has a more diverse ichthyofauna and shows some evidence of a former connection to nearby Lake Managua [47].

The colonization of new habitats by small, genetically homogenous founding populations is likely to promote speciation through both sympatric and allopatric mechanisms [48]. A corollary to this inference is that the particular suite of alleles present in the founding population is critical to the rate of divergence and speciation. It is generally perceived that the geographic context of habitat islands determines most of a colonizing population's evolutionary trajectory. Under this model, the more isolated an island, the more likely divergence will be as a result of restricted gene flow [48]. Therefore, *F*-statistics demonstrating more divergence of the Lake Apoyo and Lake Xiloa populations than of those in Lake Masaya represent either the divergence associated with the uniqueness of the founding individuals, different mechanisms of gene flow between the lakes (e.g. more human-mediated dispersal to Lake Masaya), or environmental factors such as variation in the frequency and magnitude of volcanic activity, that is unique to each of the crater lakes.

It is difficult to distinguish between demographic factors, such as population size and migration rate, from selective pressures that result in evolutionary change. But it is the combination of these factors that is most relevant to studies of speciation as they can influence the strength and direction of each other in striking ways. These results do not detect any meaningful trends in genetic diversity during a 16-year period. As a conclusion, the evolutionary forces driving speciation in places such as Lake Apoyo are persistent and not likely to be heavily influenced by demographic changes over a few (~20) generations. This persistence of evolutionary forces is apparent despite ongoing anthropogenic pressures on the lakes, including increased fishing and the introduction of non-native competitors [22], as well as any changes expected within the small crater populations due to stochastic or abiotic factors. In sum, these results further support the previous inferences [3-5], based on different collections of the fish in different years, of sympatric and allopatric speciation in these lakes.

Importantly for researchers employing population genetic analyses for the inference of evolutionary process, these results indicate that variation in sample collection design do not necessarily bias the conclusions drawn on limited data. Further investigation of what aspects of sampling design and field collection of data, including sampling the individuals as well as the particular genetic markers used, will help clarify the best collection strategy for guaranteeing robust inferences of evolutionary processes from population genetic data.

## Authors' contributions

PMEB helped collect the molecular data, designed and performed the analyses, and drafted the manuscript. MB participated in sample collection and helped collect the molecular data. AM conceived of the study, participated in sample collection, and helped draft the manuscript. All authors have read and approved of the final manuscript.
